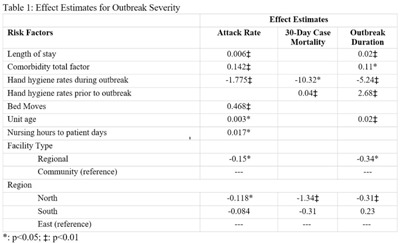# Risk factors associated with SARS-CoV-2 transmission, outbreak duration, and mortality in Fraser Health acute-care settings

**DOI:** 10.1017/ash.2022.129

**Published:** 2022-05-16

**Authors:** Tamara Duncombe, Matthew Garrod, Wang Xuetao, Joyce Ng, Eunsun Lee, Katy Short, Kennard Tan

## Abstract

**Background:** Transmission of SARS-CoV-2 in acute-care settings affects patients, healthcare workers, and the already-burdened healthcare system. An analysis of risk factors associated with outbreak severity was conducted to inform prevention strategies. **Methods:** This study was a cross-sectional analysis of COVID-19 outbreaks at Fraser Health (FH) acute-care sites between March 2020 and March 2021. Outbreak severity measures included COVID-19 attack rate, outbreak duration, and 30-day case mortality. Covariates at patient, outbreak, unit level, and facility level were included (Table 1). Generalized linear models with generalized estimation equations were used for all outcome measures, with outbreak duration and 30-day case mortality using multivariate negative binomial distributions, and attack rate using Gaussian distribution. A *P* value of .05 indicated statistical significance. Analyses

were performed using SAS version 3.8 software, R version 4.1.0 software, and Stata version 16.0 software. **Results:** Between March 2020 and March 2021, 54 COVID-19 outbreaks were declared in FH acute-care sites involving 455 SARS-CoV-2–positive patients. The average outbreak duration was 23 days, the average attack rate was 28%, and the average 30-day all-cause mortality per outbreak was 2 deaths. The results of the full models are shown in Table 1. **Discussion:** We identified an inverse relationship between increased hand hygiene compliance during outbreaks and all 3 severity measures. Paradoxically, hand hygiene rates in the year prior to the pandemic were positively associated with duration and mortality. Increased unit age was also associated with increases in each of the severity measures. Comorbidity total factor was correlated with outbreak attack rate and duration, demonstrating the importance of individual patient characteristics in an outbreak. **Conclusions:** Our findings highlight the importance of hand hygiene practices during an outbreak. Additionally, it is important to understand the difficulties faced by older facilities, many of which face infrastructural challenges. This study reinforces the need to incorporate infection control standards into healthcare planning and construction.

**Funding:** None

**Disclosures:** None

**Table:**